# Exploring the histogenesis of STK11 adnexal tumour using electron microscopy

**DOI:** 10.1007/s00428-024-03763-2

**Published:** 2024-02-20

**Authors:** Nuria Mascaro, Lamia Sabry Aboelnasr, Motasim Masood, Ernesto Yague, Linda Moran, Mona El-Bahrawy

**Affiliations:** 1 Department of Metabolism, Digestion and Reproduction, Imperial College London, Hammersmith Campus, DuCane Road, London, W12 0NN UK; 2 Department of Cancer and Surgery, Imperial College London, London, UK; 3 Electron Microscopy Unit, Imperial College Healthcare NHS Trust, London, UK; 4 Department of Pathology, Faculty of Medicine, University of Alexandria, Alexandria, Egypt

**Keywords:** STK11 adnexal tumour, Peutz-Jeghers syndrome, Electron microscopy

## Abstract

STK11 adnexal tumour is a recently described female genital tract tumour, usually identified in a paratubal location, often associated with Peutz-Jeghers syndrome (PJS) and with *STK11* gene alterations identified in most of the cases. Morphologically, this tumour is composed of cells arranged in a variety of patterns, including cords, trabeculae, tubules and cystic and acinar structures. The cells are only moderately pleomorphic and mitotic activity is variable. As tumour cells express epithelial, sex cord stromal and mesothelial markers, STK11 adnexal tumour may be of sex cord stromal, epithelial or mesothelial origin; a Wolffian origin has also been suggested. We report the ultrastructural features of two STK11 adnexal tumours and compare their ultrastructural features with those of other sex cord stromal tumours, a granulosa cell tumour cell line, as well as the known ultrastructural features of epithelial, mesothelial and Wolffian cells. On ultrastructural examination, two STK11 adnexal tumours showed an admixture of elongated cells with regular elongated nuclei and polygonal cells with nuclei showing markedly irregular outlines and prominent nucleoli. Extracellular collagen fibres were identified. These are common ultrastructural features of sex cord stromal tumours, principally sex cord tumour with annular tubules; no ultrastructural features of epithelial, mesothelial or Wolffian cells were found. These findings in conjunction with the shared clinical and genetic association with PJS and shared molecular changes in *STK11* gene suggest that STK11 adnexal tumour represents a poorly differentiated sex cord tumour.

## Introduction

There is a well-documented relationship between some ovarian neoplasms including sex cord stromal tumours (SCSTs) with hereditary diseases such as Peutz-Jeghers syndrome (PJS), a rare autosomal dominant genetic condition associated with an increased risk of developing intestinal lesions and other tumours. There have been several reports of adnexal tumours detected mainly in patients suffering from PJS, and occasionally in patients not suffering from PJS, which did not conform with any existing described tumour types, and had previously been classified in the sex cord stromal or Wolffian tumour families [1–3]. Bennett et al. studied the morphological, clinicopathological and molecular features of 22 cases of this tumour. The tumour appeared to be linked to PJS, as half of the reported patients had the syndrome. The authors concluded that this neoplasm is distinctive and is of uncertain histogenesis and designated it STK11 adnexal tumour [4].

The histogenesis of this tumour remains uncertain as the morphology studied by light microscopy and the immunoprofile suggest the tumour could potentially be of sex cord stromal, epithelial, mesothelial or Wolffian origin. Electron microscopy (EM) is a valuable tool for studying the ultrastructure of tissue and cell compartments in much more detail than light microscopy. In this study, the ultrastructural features of two STK11 adnexal tumours were investigated with EM to explore their histogenesis. The identified ultrastructural features were compared to those studied in other SCSTs, and an adult-type granulosa cell tumour cell line, as well as with known ultrastructural features of epithelial and mesothelial cells.

## Materials and methods

### Tumour samples

The study included formalin-fixed paraffin-embedded (FFPE) tissue from 2 cases of STK11 adnexal tumours and 4 cases of other types of SCSTs, including SCTAT (1 case), adult type granulosa cell tumour (AGCT, 2 cases) and fibroma (1 case). Tumour samples were obtained from the Department of Histopathology at Imperial College Healthcare NHS Trust.

### Cell line

KGN human adult-type granulosa cell tumour cell line was obtained from the Japanese Collection of Research Bioresources Cell Bank (Japan).

### Methods

#### Tissue culture: cell maintenance

KGN cells were cultured in Dulbecco’s Modified Eagle Medium (DMEM) 1X with GlutaMax Supplement and sodium pyruvate (Gibco, Cat No. 10567-014, Lot No. 2458359) low in D-glucose (1 g/L), 1% penicillin-streptomycin solution and 10% foetal calf serum (FCS), at 37 °C, 5% CO_2_. At 70% confluence, the cells were harvested by trypsin digestion and either plated again for further growth or fixed in standard EM fixative (3% glutaraldehyde in 0.1M sodium cacodylate buffer (pH 7.4). The cell pellets were processed for EM examination at the Electron Microscopy Unit at Imperial College Healthcare NHS Trust.

#### Tumour tissue selection and processing for electron microscopy

Samples were processed for EM examination at the Electron Microscopy Unit at Imperial College Healthcare NHS Trust. Formalin-fixed paraffin-embedded tumour tissue blocks were selected, and H&E-stained tissue sections were examined to select areas for ultrastructural examination. The selected tissue was then dewaxed and reprocessed in resin. Ultrathin sections cut from the samples were mounted on EM grids. EM grids were loaded into a Hitachi H-7685 transmission electron microscope (TEM).

Using TEM, the following features were studied: cell shape, nuclear shape, nuclear membrane regularity, chromatin pattern, nucleoli, cytoplasmic organelles and structures and extracellular fibres. Digital images were taken at different magnifications.

The features of the different tumours and the cell line were compared to one another and to the ultrastructural features of epithelial, mesothelial and Wolffian cells that are well described in the literature.

## Results

### Clinicopathologic features

**Fig. 1:**
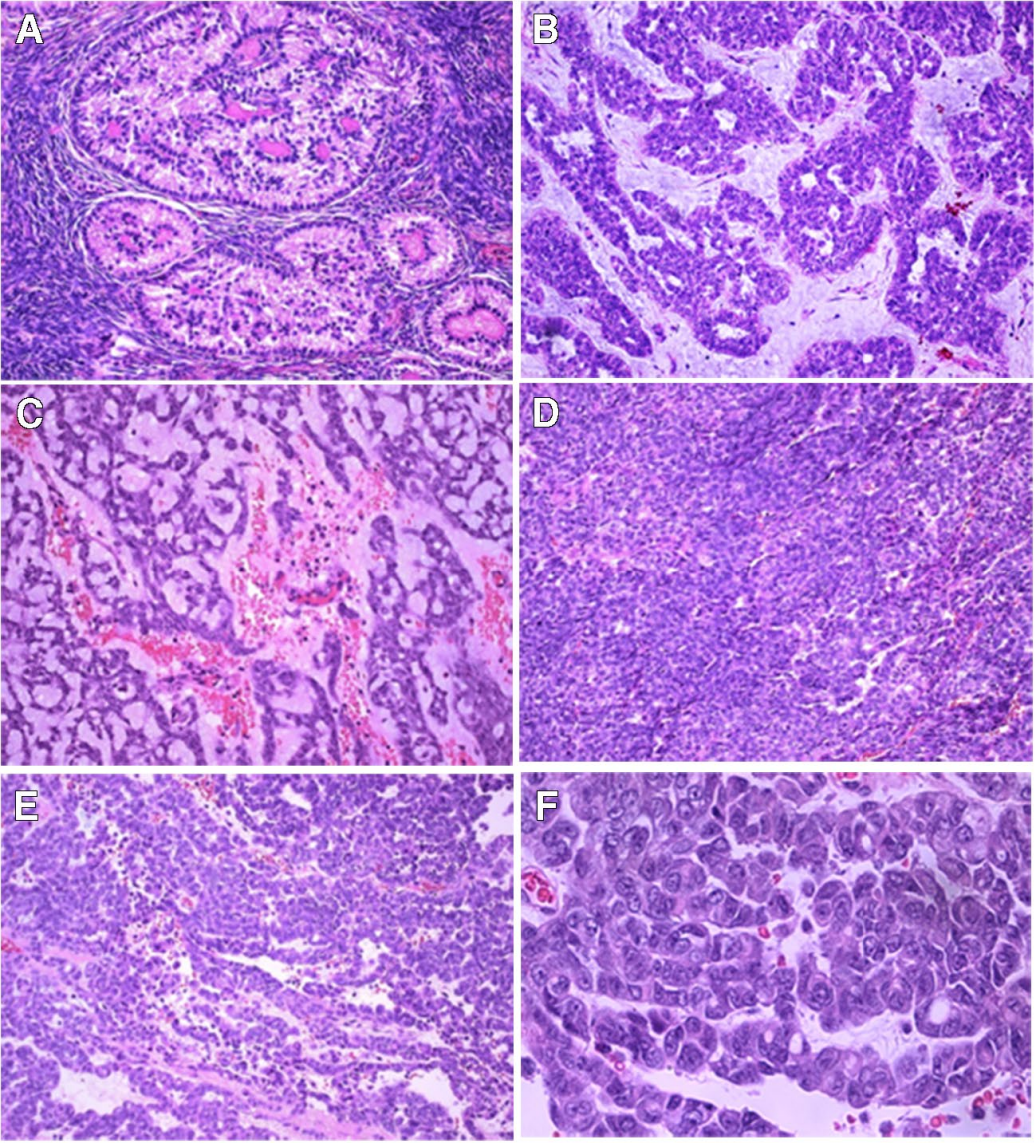
Histological features of SCTAT (**A** X100) and STK11 adnexal tumour (**B**–**F**); the latter showing glandular (**B** X100) and trabecular (**C** X200) formation within a myxoid stroma, solid (**D** X100) and corded (**E** X200) architecture. The cells have little cytoplasm and enlarged nuclei with conspicuous nucleoli and moderate pleomorphism (**F** X 400)

Case 1: A PJS patient presented at 45 years of age with a neoplasm invading the wall of the fallopian tube, and disseminated in the abdominal cavity, and pelvis. The tumour tissue from the adnexal mass and the metastatic tumour nodules was solid with haemorrhagic areas. Microscopically, the tumour cells were arranged in ill-formed pseudoacinar structures, trabeculae, small clusters and cords set in myxoid stroma with areas of hyalinisation. The cells had pale eosinophilic cytoplasm and showed oval nuclei with conspicuous nucleoli (Fig. 1). Occasional cells showed nuclear grooves. There were frequent mitotic figures (5/10HPFs) and moderate nuclear pleomorphism.

On immunostaining, the tumour cells showed strong expression of calretinin, inhibin, vimentin, WT-1 and oestrogen receptor (ER) and were negative for cytokeratin 7 (CK7), and epithelial membrane antigen (EMA).

Subsequently, the patient had total abdominal hysterectomy with bilateral salpingooopherectomy. In one ovary, a SCTAT was identified. The patient had subsequent repeated recurrences of the adnexal malignant tumour.

#### Genetic testing

Targeted sequencing showed *STK11* missense mutation with a single base-pair change (c.487G>C; p.Gly163Arg) in exon 4.

Case 2: The patient who does not have PJS presented at 30 years of age with disseminated abdominal tumour in the pouch of Douglas, pelvic sidewall, both ovaries and invading the wall of one fallopian tube. The tumour tissue from all sites showed variable architecture including cords, tubules, papillae and cystic structures. The tumour was composed of small cuboidal polygonal cells with little cytoplasm and irregular nuclei showing nuclear grooves. The nuclei were vesicular with conspicuous nucleoli. Mitoses were brisk. Some areas showed spindle cells. On immunostaining, the tumour cells expressed inhibin, calretinin, vimentin, ER, WT1 and CK7 and were negative for EMA.

The patient had a TAH and BSO and both ovaries contained a SCTAT on microscopic examination, which on the right side appeared to abut the surface of the ovary and focally extend to periadnexal tissue. On the surface of the right ovary, there was a deposit of tumour with similar features to the original malignant pelvic tumour. The patient experienced multiple subsequent recurrences.

#### Genetic testing

Targeted sequencing showed a deletion in *STK11* in the region of Exons 1-9.

Figure 1 shows histological features of STK11 adnexal tumour and SCTAT.

### Ultrastructural features

#### STK11 adnexal tumour ultrastructural features

**Fig. 2:**
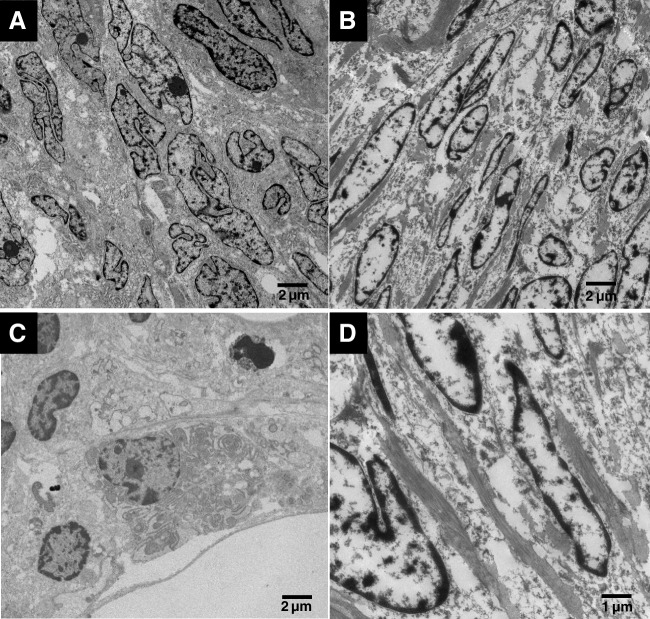
Ultrastructural features of STK11 tumour. There are polygonal cells with markedly irregular nuclei with irregularly distributed chromatin (**A**) and elongated cells showing margination of chromatin (**B**). The cells are rich in cytoplasmic organelles, mainly endoplasmic reticulum (**C**). There are intercellular collagen bundles (**D**)

Both STK11 adnexal tumours showed similar ultrastructural features. Two types of cells were identified, including elongated-shaped cells with regular elongated nuclei and polygonal-shaped cells with nuclei showing irregular outlines of the nuclear membrane. The nucleo-cytoplasmic ratio was generally high. Some of the nuclei of the round cells showed nucleoli, some nuclei had chromatin distributed at the periphery, some had uniformly distributed chromatin while others showed chromatin clumps surrounding the nucleoli. The cytoplasm of the round cells was rich in organelles, mainly rough endoplasmic reticulum (RER). Collagen filaments were present between cells (Fig. 2).

#### KGN cell line ultrastructural features

**Fig. 3:**
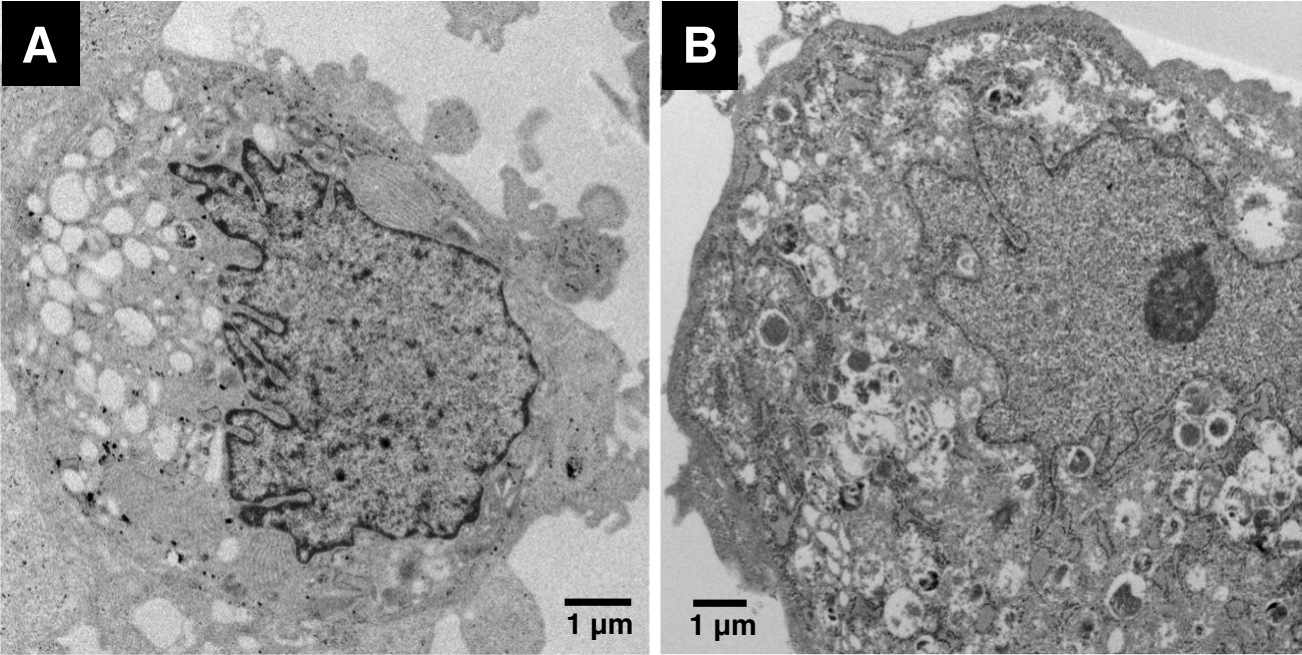
Ultrastructural features of the adult-type granulosa cell tumour cell line, KGN. The cells are polygonal and show nuclei with markedly irregular outlines (**A**) and cytoplasm rich in organelles (**B**)

KGN cells showed nuclei with irregular nuclear membranes with many invaginations and containing prominent nucleoli. The chromatin distribution was mainly uniform, with some margination at the periphery of the nucleus. The cytoplasm was rich in organelles (Fig. 3).

#### Ultrastructural features of other SCST types

The ultrastructural features of 3 SCSTs were studied, including a SCTAT, AGCT and fibroma.

**Fig. 4:**
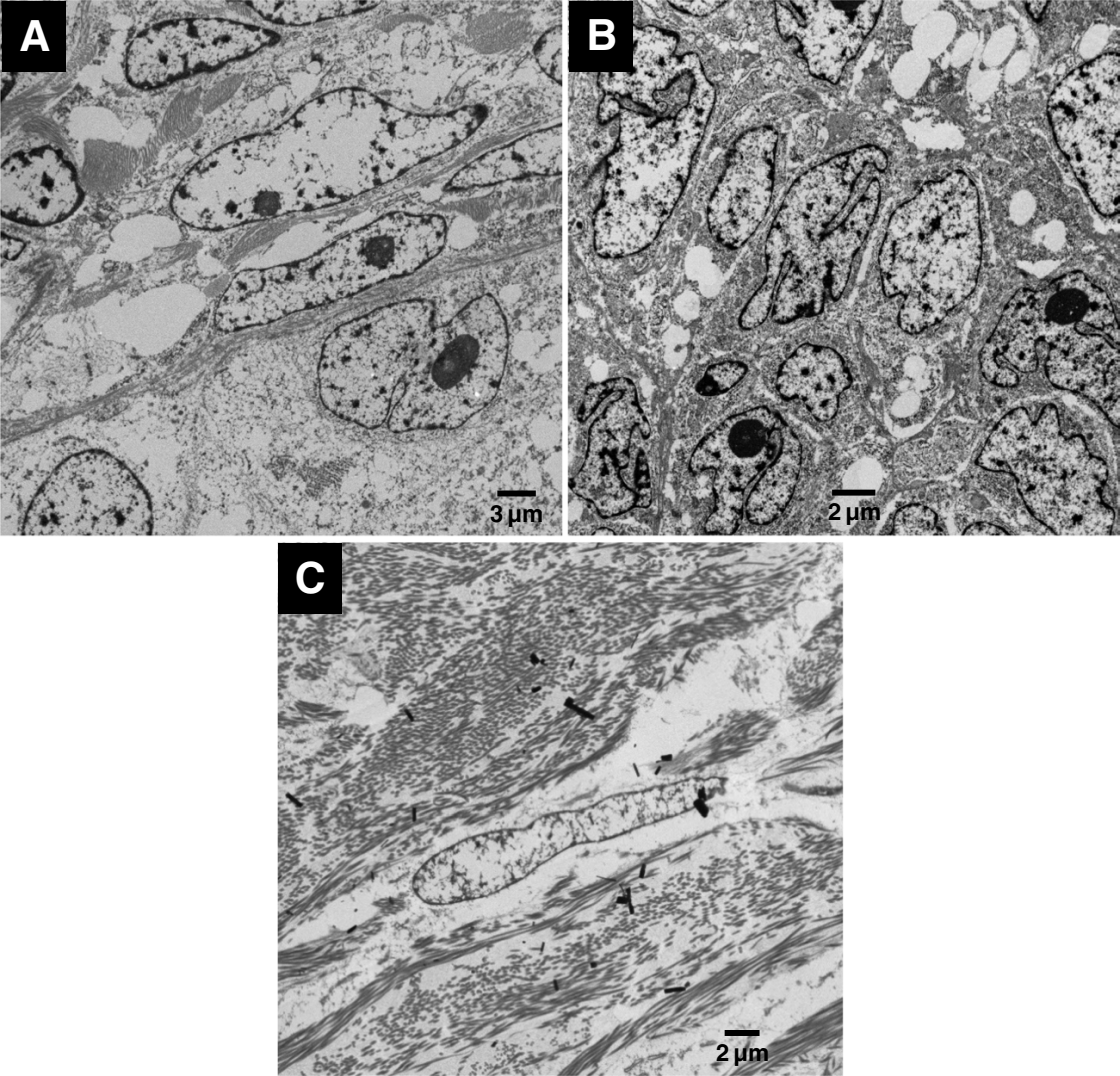
Ultrastructural features of other SCST. **A** SCTAT shows both polygonal cells with irregular nuclei featuring conspicuous nucleoli and elongated cells. There are collagen bundles between cells. **B** Granulosa cell tumour shows polygonal cells with irregular nuclear membrane, some showing prominent nucleoli. **C** Fibroma includes elongated cells with extensive presence of collagen bundles in the stroma

The SCTAT showed two types of cells, including elongated and polygonal cells with irregular nuclear outlines and prominent nucleoli. The nuclei of the round to polygonal cells had irregular outlines and the chromatin was distributed at the periphery and also in clumps through the nucleus. Collagen bundles were seen between cells (Fig. 4A).

Granulosa cell tumour cells from the 2 studied tumours were polygonal with nuclei showing irregular nuclear outlines with many invaginations. Nucleoli were present in some cells and the chromatin was mainly distributed in clumps. The cytoplasm was rich in organelles (Fig. 4B).

Fibroma cells were elongated with the nuclei showing uniform distribution of chromatin throughout the nucleus. There was a notable content of collagen fibres in the stroma (Fig. 4C).

## Discussion

STK11 adnexal tumours appear to arise paratubal, within the fallopian tube, ovary, abdominopelvic soft tissue and frequently metastasise in the pelvis and abdomen [4]. Morphologically, these tumours are usually cellular and show various patterns, including anastomosing cords and trabeculae, as well as tubular, cystic, cribriform and microacinar formations. The stroma shows variable cellularity and may show myxoid change, oedema and hyalinisation. The tumour cells are mainly columnar to cuboidal cells, but a spindled morphology can be seen. The tumour cell cytoplasm ranges from eosinophilic, to finely vacuolated, to clear. Nuclei are round to ovoid, with vesicular chromatin and often prominent nucleoli. Nuclei have irregular and angulated contours, with some showing nuclear grooves. Mitoses range from 2 to 20 per 10 high-power fields. SCTATs are noted in the ovaries of some cases [4]. The cells show positivity for a wide range of epithelial, sex cord and mesothelial markers. The tumour cells show variable expression of cytokeratins (e.g. CAM5.2, AE1/AE3, cytokeratin 7), EMA, inhibin, calretinin, SF1, WT-1, AR, ER, PR, PAX8, CD10 and D2-40, and are negative for claudin-4 and FOXL2. *STK11* gene alterations are identified in 99% of tumours, including mutations and deletions [4].

The two STK11 tumours included in this study showed the histological features, immunoprofile, genetic alterations and clinical behaviour reported for these tumours. One of the cases was originally reported by us as a case report of a SCTAT in a PJS patient showing unusual features and biological behaviour [1], and also included in the case series describing this tumour [4]. The histogenesis of STK11 adnexal tumour is not completely clear, and origins from sex cord stromal, epithelial or mesothelial or Wollfian lineage have been proposed. Based on the clinicopathological, morphological, immunophenotypic and molecular characteristics, we hypothesised that these tumours are of sex cord stromal lineage.

To test this hypothesis, and in addition to the 2 STK 11 tumours, we included in the study 2 AGCTs, and a SCTAT, which are types of sex cord tumours, and a fibroma, which is a type of stromal tumours. Based on the examination of multiple haematoxylin and eosin–stained sections from the different tumour types, the histological diagnosis was determined on the basis of histological features. Finding distinctly different ultrastructural features supported the fact that they are different diagnostic entities. While SCTAT and STK11 adnexal tumours shared the presence of the 2 types of cells on ultrastructural examination, on histological examination, STK11 adnexal tumour lacked the characteristic histological architecture of SCTAT. Table 1 summarises the histological features, immunoprofile and ultrastructural features of the different tumour types included in this study.

**Table 1:** Histological features, immunoprofile and ultrastructural features of STK11 tumour, sex cord tumour with annular tubules (SCTSAT), adult granulosa cell tumour (AGCT) and fibroma [4, 5].

Tumour type	Morphology	Immunoprofile	Ultrastructural features
STK11 adnexal tumour	Tumours are usually cellular and show various patterns, including anastomosing cords and trabeculae, as well as tubular, cystic, cribriform and microacinar formations. The stroma shows variable cellularity and may show myxoid change, oedema and hyalinisation. The tumour cells are mainly columnar to cuboidal cells, but a spindled morphology can be seen. The tumour cell cytoplasm ranges from eosinophilic, to finely vacuolated, to clear. Nuclei are round to ovoid, with vesicular chromatin and often prominent nucleoli. Nuclei have irregular, and angulated contours, with some showing nuclear grooves.	The tumour cells show variable expression of cytokeratins (e.g. CAM5.2, AE1/AE3, cytokeratin 7), EMA, inhibin, calretinin, SF1, WT-1, AR, ER, PR, PAX8, CD10, D2-40, and are negative for claudin-4, FOXL2.	Two types of cells are identified, including elongated cells with regular elongated nuclei and polygonal-shaped cells with nuclei showing irregular outlines of the nuclear membrane. Some of the nuclei of the round cells show nucleoli, some nuclei have chromatin distributed at the periphery, some have uniformly distributed chromatin while others show chromatin clumps surrounding the nucleoli. The cytoplasm of the round cells is rich in organelles, mainly rough endoplasmic reticulum (RER). Collagen filaments are present between cells.
SCTAT	Tumours show rounded nests composed of tubules formed of elongated cells encircling hyaline basement membrane-like material. There may be areas with solid proliferation of granulosa or Sertoli cell–like morphology.	The cells express inhibin, calretinin, FOXL2, CD56, SF1 and WT1 and are negative for EMA.	Two types of cells are present, including elongated and polygonal cells with irregular nuclear outlines and prominent nucleoli. The nuclei of the round to polygonal cells have irregular outlines and the chromatin is distributed at the periphery and also in clumps through the nucleus. Collagen bundles are seen between cells.
AGCT	Tumours can show variable architectural patterns, including diffuse, corded, trabeculated, insular, microfollicular (Call-Exener bodies), macrofollicular, pseudopapillary, gyriform and sarcomatoid. The cells are usually polygonal, with nuclei showing nuclear grooves.	Cells show variable expression of inhibin, calretinin, FOXL2, and SF1, ER, WT1. Cells can express pancytokeratins, but are negative for EMA.	Polygonal cells with nuclei showing irregular nuclear outlines with many invaginations. Nucleoli are present in some cells and the chromatin is mainly distributed in clumps. The cytoplasm is rich in organelles.
Fibroma	Intersecting fascicles of spindled cells within variably collagenous stroma.	Cells show variable expression of inhibin, calretinin, SF1, WT1, CD56, ER and PR.	Elongated cells with the nuclei showing uniform distribution of chromatin throughout the nucleus. There is a notable content of collagen fibres in the stroma.

STK11 adnexal tumours may be considered epithelial type due to the acinar and tubular formation, as well as the expression of broad-spectrum cytokeratin. However, *STK11* alterations are virtually non-existent in adnexal mullerian adenocarcinomas. The variable architectural patterns, location and expression of calretinin bring mesothelioma in the differential diagnosis. This is further supported by the expression of WT1, calretinin and D2–40, and rarely malignant mesotheliomas may harbour *STK11* alterations [6]. However, STK11 adnexal tumours usually form a discrete mass in contrast to the more diffuse or plaque-like growth frequent to mesotheliomas [7]. Also, hormone receptors commonly expressed by STK11 adnexal tumours are uncommonly expressed in mesotheliomas [8].

On ultrastructural examination, epithelial cells are characterised by well-formed cell-cell junctions such as tight junctions and possibly microvilli in the apical part of the cells. Mesothelial cells are normally elongated, flattened, squamous-like cells with well-developed cell-cell junctions [9]. Perinuclear tonofilaments, elongated and fragmented microvilli and giant desmosomes are some of the structures seen in these cells [10]. Wolffian cells show tight junctions, desmosomes and prominent microvilli on the cell surface [11].

In our study, none of the typical ultrastructural features of epithelial cells, mesothelial cells or Wolffian cells is identified in the tumour cells. The ultrastructural features most resembled those of SCTAT. Both tumours contained two types of cells, namely the polygonal cells with nuclei featuring irregular outlines, heterogeneous chromatin distribution and nucleoli and the elongated cells with elongated nuclei featuring fine uniformly distributed chromatin. The granulosa cell tumour also showed a resemblance to STK11 adnexal tumour as it was composed of cells similar to the polygonal cells seen in STK11 tumour. These features were seen in the cells of the granulosa cell tumours studied as well as the cells of the granulosa cell tumour cell line KGN. Studying an *in vitro* model of AGCT offered the opportunity to study the ultrastructural features of individual cells in detail and finding the cells showed similar features to the tumour cells processed from fixed tumour tissue from the 2 AGCT—namely the polygonal cells with nuclei featuring a very irregular nuclear contour and similar chromatin distribution—was reassuring of the specificity of the features to granulosa cell tumour.

The ultrastructural features of STK11 adnexal tumour have not been previously described. In this study, we explored the ultrastructural features of two STK11 adnexal tumour cases in comparison to those of other SCSTs, a granulosa cell tumour cell line and the well-described ultrastructural features of epithelial and mesothelial cells. The cells of STK11 adnexal tumours showed none of the characteristic features of epithelial cells or mesothelial cells, making it unlikely that these tumours are of epithelial or mesothelial histogenesis. On the other hand, two types of SCST subtypes showed a clear resemblance of their ultrastructural features to those of STK11 adnexal tumour. The two cell types identified in STK11 adnexal tumour were almost identical to those identified in SCTAT, while one of the two cell types was almost identical to those identified in granulosa cell tumour and the granulosa cell tumour cell line KGN; all cells of sex cord type. However, there were distinct differences from fibroma, a tumour of stromal histogenesis, which only included elongated cells that showed more spindle-shaped nuclei compared to the elongated cells seen in STK11 adnexal tumour and SCTAT. In addition, the amount of collagen fibres seen in fibroma was distinctly more abundant as compared to the other tumours.

This further suggests sex cord rather than stromal differentiation of STK11 adnexal tumour.

STK11 adnexal tumours are usually present in an adnexal location and show diverse architectural patterns, a common association with PJS and recurrent *STK11* alteration. While the use of electron microscopy is not a modality that would need to be used for the clinical diagnosis of these tumours in routine practice, studying the ultrastructure of the tumours provides additional information for determining the histogenesis of these tumours. The tumours show ultrastructural features most similar to those of SCTAT suggesting they represent a poorly differentiated sex cord tumour, most reminiscent of a poorly differentiated/dedifferentiated SCTAT. Our hypothesis is STK11 adnexal tumour is a sex cord stromal tumour and may represent a dedifferentiated/poorly differentiated SCTAT is based on the fact that they share the same molecular aberration and that some cases of STK11 adenxal tumour are associated with SCTAT synchronously identified or seen in earlier or subsequent ovarian biopsies for the patient. The fact that at the ultrastructural level they share an identical cellular composition tends to support this hypothesis. The fact that many of these tumours may present in extraovarian locations is not against their histogenesis of sex cord origin as sex cord stromal tumours can also arise in extraovarian locations [3, 11, 12].

## Data Availability

All data generated or analysed during this study are included in this published article.

## References

[1] Barker D, Sharma R, McIndoe A et al (2010) An unusual case of sex cord tumor with annular tubules with malignant transformation in a patient with Peutz-Jeghers syndrome. Int J Gynecol Pathol 29(1):27–32 19952941 10.1097/PGP.0b013e3181b6a7c2

[2] Bennett JA, Ritterhouse LL, Furtado LV et al (2020) Female adnexal tumors of probable Wolffian origin: morphological, immunohistochemical, and molecular analysis of 15 cases. Mod Pathol 33(4):734–747 31591497 10.1038/s41379-019-0375-9

[3] Simpson JL, Michael H, Roth LM (1998) Unclassified sex cord-stromal tumors of the ovary: a report of eight cases. Arch Pathol Lab Med 122(1):52–55 9448017

[4] Bennett JA, Young RH, Howitt BE et al (2021) A distinctive adnexal (usually paratubal) neoplasm often associated with Peutz-Jeghers syndrome and characterized by STK11 alterations (STK11 adnexal tumor): a report of 22 cases. Am J Surg Pathol 45(8):1061–1074 33534223 10.1097/PAS.0000000000001677 PMC8277663

[5] WHO (2020) Classification of tumours. Female genital tract. International Agency for Cancer

[6] Ugurluer G, Chang K, Gamez ME et al (2016) Genome-based mutational analysis by next generation sequencing in patients with malignant pleural and peritoneal mesothelioma. Anticancer Res 36(5):2331–2338 27127140

[7] Marchevsky AM, Khoor A, Walts AE et al (2020) Localized malignant mesothelioma, an unusual and poorly characterized neoplasm of serosal origin: best current evidence from the literature and the International Mesothelioma Panel. Mod Pathol 33(2):281–296 31485011 10.1038/s41379-019-0352-3 PMC10428660

[8] Chapel DB, Schulte JJ, Husain AN, Krausz T (2020) Application of immunohistochemistry in diagnosis and management of malignant mesothelioma. Transl Lung Cancer Res 9(Suppl 1):S3–S27 32206567 10.21037/tlcr.2019.11.29 PMC7082260

[9] Mutsaers SE (2002) Mesothelial cells: their structure, function and role in serosal repair. Respirology 7(3):171–191 12153683 10.1046/j.1440-1843.2002.00404.x

[10] Oczypok EA, Oury TD (2015) Electron microscopy remains the gold standard for the diagnosis of epithelial malignant mesothelioma: a case study. Ultrastruct Pathol 39(2):153–158 25268063 10.3109/01913123.2014.960542 PMC4379130

[11] Shah R, McCluggage WG (2017) Unclassifiable malignant extraovarian sex cord-stromal tumors: report of 3 cases and review of extraovarian sex cord-stromal tumors. Int J Gynecol Pathol 36(5):438–446 27801763 10.1097/PGP.0000000000000350

[12] Wang J, Papanastasopoulos P, Savage P, Smith JR, Fisher C, El-Bahrawy MA (2015) A unique case of extraovarian sex-cord stromal fibrosarcoma, with subsequent relapse of differentiated sex-cord tumor. Int J Gynecol Pathol 34(4):363–368 25760903 10.1097/PGP.0000000000000151

